# Transmission of Two Viruses that Cause Barley Yellow Dwarf is Controlled by Different Loci in the Aphid, *Schizaphis graminum*

**DOI:** 10.1673/031.007.2501

**Published:** 2007-04-19

**Authors:** Stewart M. Gray, Marina C Caillaud, Mary Burrows, Dawn M. Smith

**Affiliations:** ^1^ USDA, ARS; ^2^ Department of Plant Pathology, Cornell University, Ithaca, NY 14853; ^3^ Department of Biological Sciences, Ithaca College, Ithaca, NY 14850

**Keywords:** polerovirus, luteovirus, greenbug, vector competence, circulative, persistent transmission

## Abstract

Clonal populations of the aphid, *Schizaphis graminum*, have been separated into biotypes based on host preference and their ability to overcome resistance genes in wheat. Recently, several biotypes were found to differ in their ability to transmit one or more of the viruses that cause barley yellow dwarf disease in grain crops, and vector competence was linked to host preference. The genetics of host preference has been studied in *S. graminum*, but how this may relate to the transmission of plant viruses is unknown. Sexual morphs of a vector and nonvector *S. graminum* genotype were induced from parthenogenetic females and reciprocal crosses made. Eighty-nine hybrids were generated and maintained by parthenogenesis. Each hybrid was evaluated for its ability to transmit *Barley yellow dwarf virus-PAV and Cereal yellow dwarf virus-RPV*, and for its ability to colonize two wheat genotypes each expressing a different gene that confers resistance to *S. graminum*. The F1 genotypes were genetically variable for their ability to transmit virus and to colonize the aphid resistant wheat, but these traits were not genetically correlated. Individual F1 genotypes ranged in transmission efficiency from 0–100% for both viruses, although the overall mean transmission efficiency was similar to the transmission competent parent, indicating directional dominance. The direction of the cross did not significantly affect the vector competency for either virus, suggesting that maternally inherited cytoplasmic factors, or bacterial endosymbionts, did not contribute significantly to the inheritance of vector competency in *S. graminum*. Importantly, there was no genetic correlation between the ability to transmit *Barley yellow dwarf virus* and *Cereal yellow dwarf virus-RPV* in the F1 genotypes. These results taken together indicate that multiple loci are involved in the circulative transmission, and that the successful transmission of these closely related viruses is regulated by different sets of aphid genes.

## Introduction

The molecular and cellular basis of virus-vector interactions that regulate transmission are not well understood, but it is clear that genetic elements within both the virus and the vector ultimately determine if a particular species or individual within a species of arthropod is able to transmit a particular virus strain (Gray and Banerjee 1999). Environmental or abiotic factors also play a role in determining virus-vector interactions, but in general, these factors seem to influence the efficiency of the interaction rather than prevent the interaction (Power and Gray 1995; Weaver 1997). A majority of arthropod-borne plant and animal viruses that circulate in their insect vectors, and in many cases infect the insect, are transmitted by one or a limited number of closely related vector species. Furthermore, populations within a species will differ in their ability to efficiently vector certain viruses. Intraspecific variation in vector capacity has been described for circulative plant viruses (Bencharki et al. 2000; Gray 1999) and animal viruses (Gooding 1996).

Multiple studies with animal and plant viruses have inferred that transmission competence is genetically regulated in the insect vector. Indirect evidence is available from studies that characterize insect populations with different host adaptations or that are geographically isolated, and that differ in their ability to transmit various circulative viruses (Bourdin et al. 1998; Gray et al. 2002; Tardieux et al. 1990; Tesh et al. 1976). Direct evidence for genetic regulation was obtained from matings of arbovirus-competent and refractory vector genotypes and the subsequent analysis of their progeny (Beerntsen et al. 2000; Bosio et al. 2000; Failloux et al. 1999). Polygenic inheritance was inferred in several instances (Tabachnick 1994; Tardieux et al. 1991), although single locus control has been described for the susceptibility of *Culicoides variüennis* to bluetongue virus (Tabachnick 1991). Similarly, the inability of a strain of *Aedes aegypti* to transmit several flaviviruses is regulated by either a single gene or a set of closely linked genes (Miller and Mitchell 1991). Recently, quantitative trait loci have been identified in mosquitoes that regulate vector competency (Anderson et al. 2005; Bennett et al. 2005), but no insect genes have been identified that are directly linked to the regulation of arbovirus transmission.

The genetics of plant virus transmission was first studied by Storey (Storey 1932; Storey 1933). He determined that the transmission of *Maize streak virus*, a circulative, nonpropagative geminivirus, was inherited as a simple maternal-linked dominant factor in the leafhopper, *Cicadulina mbila*. Subsequent studies on the transmission of other circulative viruses by planthoppers and leafhoppers also confirmed genetic control although inheritance characteristics differed. Transmission competence of the circulative, propagative *Rice stripe virus* was recessive and not sex-linked in the planthopper, *Lagdelphax striatellus* (Kisimoto 1967). Transmission competence of the propagative *Rice hoja blanca virus* in the planthopper, *Sagosodes oryzicola*, was controlled by a single recessive gene (Zeigler and Morales 1990).

Aphids offer a unique advantage over other arthropod vectors as a model for studying the genetics of vector competence as they are cyclic parthenogens, i.e. they alternate sexual reproduction with parthenogenetic reproduction. Since no recombination occurs during parthenogenesis (Blackman 1987; Wilson et al. 2003), each parthenogenetic genotype represents a clone that can be maintained indefinitely. This allows replicate phenotypic measurements of multiple quantitative traits that increases the experimental power of any genetic analyses. The genetic integrity of various regions of the nuclear genome of an aphid clone has been verified using molecular markers (Debarro et al. 1994; Fukatsu and Ishikawa 1994; Shufran et al. 1991), although clonal fidelity is not absolute (Loxdale and Lushai 2003; Shufran et al. 2003). Nevertheless, studies have shown genetic stability for over 100 generations (Shufran et al. 1991). We have observed a 40+ year stability of transmission phenotypes in clonal populations of several aphid species for two viruses used in this study, *Barley yellow dwarf virus-PAV* (BYDV-PAV, *Luteovirus*) and *Cereal yellow dwarf virus-RPV* (CYDV-RPV, *Polerovirus*) (Power and Gray, 1995; Gray, unpublished data). These two viruses and five other related viruses cause barley yellow dwarf disease, the most economically important virus disease of cereal crops. These viruses are transmitted in a circulative-nonpropagative manner which means they must circulate through the aphid in order to be transmitted to a new plant, but they do not replicate within the aphid body. Although all aphid species tested can ingest any of these viruses if they feed on the phloem of an infected plant, each virus is transmitted only by one or a few specific species. This vector specificity is regulated by the ability of the virus to be recognized by uncharacterized receptors on either the hindgut or accessory salivary gland tissues and be transported across these tissues (see Gray and Gildow 2003 for review).

The unusual life cycle of aphids has been poorly exploited by geneticists because inducing the sexual phase and performing crosses is not possible in many species and delicate in most. However, induction of the sexual forms and crosses are now routine for some species (Birch et al. 1994; Dedryver et al. 1998; Helden and Dixon 1997; Shufran et al. 1997; Via 1992) and these genetic systems are providing valuable information on host preference, adaptation to host resistance, insecticide resistance and developmental biology. The genetics of plant virus transmission in aphids was first attempted by Bjorling and Ossiannilsson (1958). They tested the transmission phenotype of two F1 genotypes from *Myzus persicae* clones that differed in their ability to transmit *Potato leqfroll virus*. However, a limited number of progeny precluded any genetic analyses. Recently, Papura et al. (2002) tested 39 F1 progeny generated by selfing a clone of *Sitobion avenae*. The parent was an inefficient vector of BYDV-PAV, yet the transmission efficiency of the F1 progeny ranged from 0 to 88%. More recently, Dedryver et al. 2005 crossed genotypes of *S. avenae* that differed in BYDV-PAV transmission efficiency. Analysis of their F1 progeny led the authors to conclude that transmission was polygenic and inefficient transmission efficiency was the dominant phenotype. We have used another aphid, *S. graminum*, to investigate the genetics of BYDV/CYDV transmission and report here that multiple and different loci are involved in the transmission of two related virus species, BYDV-PAV and CYDV-RPV.

## Material and Methods

### Aphids

Two genotypes of *S. graminum*, originally described as biotype C (Sg-C) and biotype F (Sg-F) (Porter et al. 1997), were obtained from Dr. John Burd, USDA, ARS, Stillwater, OK. Colonies of all aphid genotypes were maintained on ‘Lud’ barley as previously described (Rochow 1969). To induce sexual morphs, aphid colonies were moved from a 22°C, 15:9 (L:D) regime to a 16°C, 11:13 (L:D) regime. Sexuals began to develop after approximately 4 weeks. Immature females (4th instars), identified by thickened hind tibia, were removed from the colony, placed in sealed containers with detached barley leaves and allowed to molt into adults, after which adult males of the other genotype were introduced into the container. Fertilized eggs (black in color) were removed from leaf pieces using a fine camel hair paint brush moistened with 1% calcium propionate and placed on sterile filter paper moistened with calcium propionate in sterile Petri plates. Eggs were incubated at 0 ± 1°C, 24 hr dark for three months after which they were transferred to 16°C, 11:13 (L:D) regime. Detached barley leaves were placed in the dish and hatching nymphs were allowed to develop on these leaves for 2 to 4 days prior to being transferred to plants, one nymph per plant. Each surviving nymph was considered as a separate genotype and the colony was maintained by parthenogenetic reproduction at 20°C, 24 hr light. New colonies of each genotype were started from three 1st instar nymphs approximately every 3 to 4 weeks (Katsar and Gray 1999).

### Virulence in wheat

The Sg-F and Sg-C biotypes were originally separated using differential cultivars of wheat possessing different resistance genes (Porter et al. 1997). Sg-F is virulent on cultivars that have the Gb2 or Gb3 gene, where Sg-C is avirulent on cultivars expressing either of these genes. Gray et al. 2002 identified that BYDV/CYDV transmission efficiency in *S. graminum* biotypes was correlated with host preference. To determine if virus transmission phenotype in the aphid was genetically linked to the virulence phenotype in wheat, the Sg-F and Sg-C parents and their F1 progeny were evaluated for their virulence on the wheat cultivars, Amigo and Largo that express the *Gb2* or *Gb3* gene, respectively. Virulence is characterized by necrotic spots with chlorotic halos that appear at feeding sites of the aphids. Avirulence is characterized by small, chlorotic spots that appear at the feeding sites, but the spots do not increase in size or become necrotic. Wheat seedlings at the two-leaf stage, three per 4” pot, were infested with approximately 20 adult aphids. Aphids were introduced onto the soil and allowed to distribute themselves on the wheat plants. The plants and aphids were enclosed in one cage and maintained at 18 ± 2°C, 16:8 (L:D) regime for 12 days. Observations of necrosis were made at 3 to 4, 7 to 8 and 11 to12 days after infestation. Damage was scored as 1, 2, 3, 4 depending on if necrosis was observed at 3 to 4 days after infestation, 7 to 8 days after infestation, 11 to 12 days after infestation or no necrosis, respectively. Experiments were repeated 2 to 4 times for each F1 genotype and approximately eight genotypes were evaluated within each experiment depending on availability of aphids. The Sg-F and Sg-C genotypes were used as controls in each experiment.

**Table 1.**
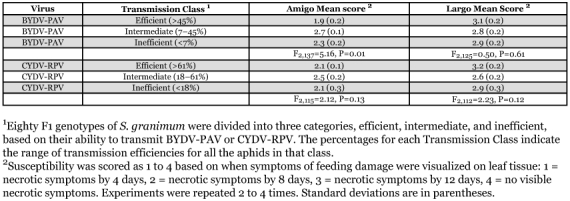
Susceptibility of two wheat cultivars to clonal lines of the aphid, *Schizaphis graminum*, that differ in their ability to transmit *Barley yellow dwarf virus-PAV or Cereal yellow dwarf virus-RPV*.

### Virus transmission assays

The virus isolates used in the study, BYDV-PAV, CYDV-RPV were previously described (Power and Gray 1995) and have been maintained in ‘Coast Black’ oat by routine transfer to young oat plants using *Rhopalosiphum padi*. During these experiments, virus source plants were inoculated by Sg-F. Virus source leaves were collected approximately 3–4 wk after inoculation. The base of the detached leaf was wrapped in cotton, moistened, and inserted into a 2 cm diameter plastic tube so that the cotton at the base of the leaf formed a plug at the end of the tube. Adult aphids were collected from a 3 week-old colony, dropped into the tube and allowed to crawl onto the leaf. A foam stopper was used to seal the top end of the tube to prevent aphids from escaping. The adults were allowed to feed on the leaf and produce young for 24 hrs prior to being removed. The nymphs were removed 48 hrs later; therefore, they were allowed a 48 to 72 hr acquisition access period depending on when they were born. This technique was used to minimize the handling of young aphids, which are easily damaged. At the end of the 48 to 72 hr acquisition access period, a majority of the nymphs had molted to second instars. Three aphids were transferred to each recipient non-infected plants (‘Coast Black’ oat) to determine transmission efficiency. Aphids were allowed a 3 to 5 day inoculation access period and then the plants were fumigated with DDVP (O,O-dimethyl-O-[2,2-dichlorovinyl phosphate) in a closed chamber, moved to a greenhouse and observed for symptom development for 3 to 5 weeks. Acquisition and inoculation access experiments were conducted in plant growth rooms maintained at 18 ± 2°C, 16:8 L:D regime. Virus transmission efficiency was calculated as the percentage of the total number of plants infested with viruliferous aphids that become infected. The transmission efficiency data were transformed as an arcsin square root prior to analysis, although the untransformed data are presented in figures. Usually each transmission experiment evaluated 8 to 10 F1 genotypes plus the two parents using 12 to 16 recipient plants for each aphid genotype. Transmission experiments were repeated 2 to 5 times for each F1 hybrid. All leaves used as virus source tissue were tested for the presence of virus using a double antibody sandwich enzyme linked immunosorbent assay (Gray et al. 1998).

### Transmission data analysis

Analysis of variance on arcsin transformed data with fixed and random factors was used to test three hypotheses regarding the mode of inheritance of virus transmission for each virus: 1) Was the transmission efficiency among the F1 genotypes genetically variable (PROC MIXED; SAS, 1990; *F1 clone* is a random factor)? 2) How did the mean transmission efficiency of all the F1 genotypes compare to the mean transmission efficiency of each parent (PROC GLM, SAS, 1990; *parent vs hybrid* is a fixed factor)? 3) Was the transmission phenotype in F1 genotypes influenced by the direction of the cross (PROC GLM; SAS, 1990; *Direction of the cross* is a fixed factor)? The means of each level of fixed effects (parents Sg-C and Sg-F; direction of the cross, Sg-F × Sg-C and Sg-C × Sg-F; mean of the F1 hybrid generation) were calculated in PROC MIXED as least-square means (LSMEANS statement), while the value for each level of the random effect (each clone within the F1 hybrid population) was estimated as a best linear unbiased predictor or BLUP (option SOLUTION). The variance components and broad-sense heritabilities (H2) were determined as the ratio between the total genetic variance and the total phenotypic variance, for each virus strain separately, by extracting between-genotypes (i.e. genetic) and environmental components of variance from one-way ANOVA's on F1 genotypes within each virus type using PROC MIXED (SAS, 1990). Genetic correlations between the transmission efficiency of the virus isolates were calculated in F1 genotypes as product-moment correlations of clone means (the rcm of (Via 1991) using PROC CORR (SAS, 1990).

**Figure 1.**
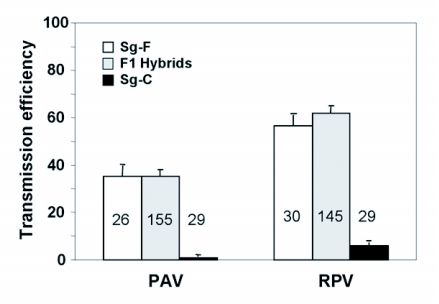
Mean transmission efficiency of BYDV-PAV and CYDV-RPV by the parental genotypes, Sg-F and Sg-C, and the F1 hybrids. Transmission efficiency was calculated as the percentage of plants infested with viruliferous aphids that became infected following a 96 hr inoculation access period. The number of recipient plants used in each experiment was 12 to 16 and the number of experiments used to calculate the means and standard errors is shown in each bar.

## Results

### Generation of Sg-F × Sg-C F1 genotypes

Twelve separate matings of Sg-F females and Sg-C males and 15 separate matings of Sg-C females and Sg-F males yielded 581 and 955 fertilized eggs, respectively. Egg hatch efficiency ranged from 49 to 56%. Due to space and resource constraints we hoped to maintain 50 F1 parthenogenetic reproducing genotypes for each cross, but only 38 and 49 genotypes survived for the Sg-F × Sg-C and Sg-C × Sg-F crosses, respectively.

### F1 segregation ratios on resistant wheat

Necrosis was evident on plants infested with Sg-F usually by 4 days with a mean damage score of 1.2. Sg-C feeding did not result in observable symptoms and a mean score of 4.0 was calculated. The reactions of the individual genotypes were generally consistent across experiments. To analyze the data, the F1 genotypes were divided into categories of inefficient vectors, intermediate vectors or efficient vectors. These divisions were somewhat arbitrary based on roughly equal numbers of hybrids in each category or where an obvious division was observed when the transmission efficiency of individual genotypes was plotted. The divisions for PAV transmission were <7%, 7–45% and >45% for inefficient, intermediate and efficient vectors, respectively. The divisions for RPV transmission were <18%, 18–61% and >61% for inefficient, intermediate and efficient vectors, respectively. The virulence reaction on ‘Amigo’ (*Gb2* gene) was significantly more severe for efficient vectors of PAV and least severe for intermediate vectors (Table 1). The same trend was evident for RPV, although the differences were not significant. There were no significant differences among vector categories for PAV or RPV and the virulence reaction on ‘Largo’ (*Gbs* gene) (Table 1).

### *S. graminum* populations segregating for virus transmission

**Figure 2.**
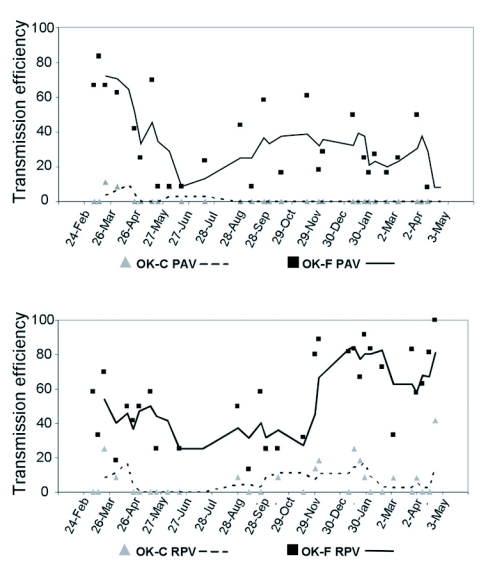
Variation in transmission efficiency of BYDV-PAV (top) and CYDV-RPV (bottom) by the parental aphids Sg-F (squares) and Sg-C (triangles) over the 14 months that the transmission efficiency experiments were conducted. The lines associated with the data points are 3-point moving averages.

The two parental *S. graminum* genotypes, Sg-F and Sg-C, behaved similarly to previous studies (Gray et al. 2002) with respect to the transmission of PAV and RPV. The two genotypes significantly differed in their ability to transmit PAV (F 1,56 = 44-55; P<0.001) and RPV (F 1,56 = 75.12; p<0.001) (Figure 1). Sg-F transmitted PAV and RPV with mean transmission efficiency of 35% and 57%, respectively, although the range was 8 to 83% and 13 to 100%, respectively. Sg-C transmitted PAV and RPV with mean transmission efficiency of 1% and 6%, respectively (Figure 1), and the range was 0 to 11% and 0 to 25%, respectively. These experiments were conducted over 14 months and transmission efficiency varied dependent upon environmental conditions. There was a general trend of lower transmission efficiency during the summer months for both PAV and RPV (Figure 2). At the end of the experiment it appears that an RPV isolate was selected that is more efficiently transmitted by Sg-F than the isolate we started with. A consistent high level of transmission of this isolate has continued in subsequent experiments (Burrows et al. 2006). In contrast, there was not a selection for a more efficiently transmitted PAV isolate.

**Figure 3.**
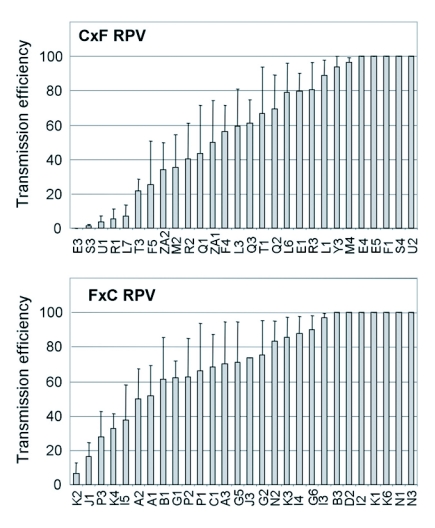
Transmission efficiency of CYDV-RPV by individual F1 genotypes (listed on the X-axis) from a cross of Sg-C females and Sg-F males (C×F) or Sg-F females and Sg-C males (F×C). The mean transmission efficiency and standard error for each F1 genotype was calculated as the percentage of plants infested with viruliferous aphids that became infected following a 96 hr inoculation access period. The number of recipient plants used in each experiment was 12 to 16 and each genotype was tested in 2 to 4 experiments.

**Figure 4.**
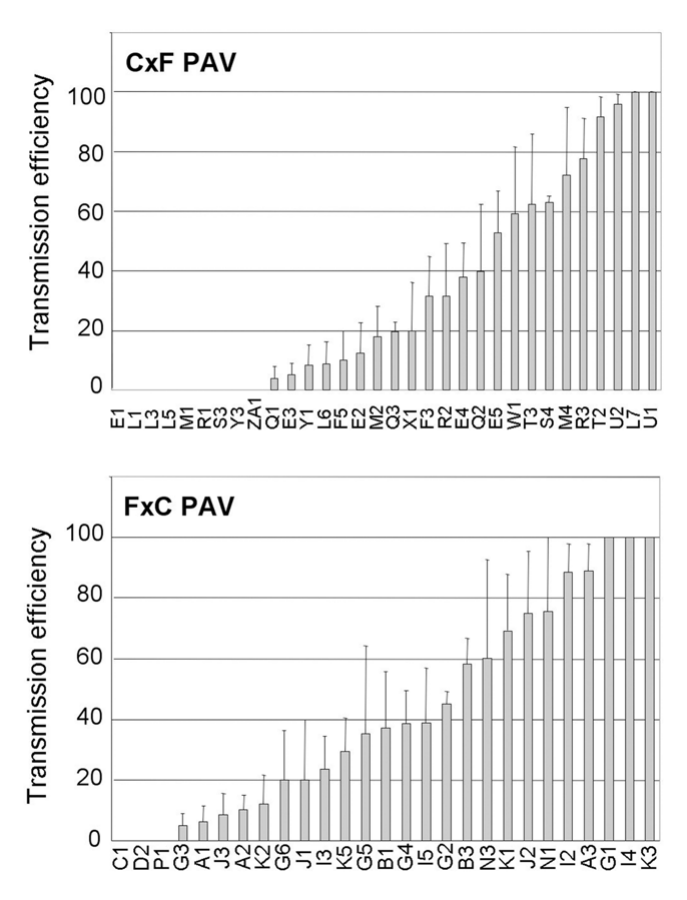
Transmission efficiency of BYDV-PAV by individual F1 genotypes (listed on the X-axis) from a cross of Sg-C females and Sg-F males (C×F) or Sg-F females and Sg-C males (F×C). The mean transmission efficiency and standard error for each F1 genotype was calculated as the percentage of plants infested with viruliferous aphids that became infected following a 96 hr inoculation access period. The number of recipient plants used in each experiment was 12 to 16 and each genotype was tested in 2 to 4 experiments.

Each of the F1 hybrid genotypes was evaluated for transmission efficiency of PAV and RPV in multiple experiments and a mean transmission efficiency was calculated. Approximately 6 to 8 F1 genotypes were tested in each experiment along with both parents that served as positive (Sg-F) and negative (Sg-C) controls in each of the F1 hybrid experiments. Due to the variation in the transmission efficiency of the parent genotypes across all experiments and a need to compare the transmission efficiencies of the F1 genotypes with each other, we calculated a corrected transmission efficiency (TE) for each F1 genotype using the following formula: TEcorr = 1-((TESgF -TEF1)/(TESgF -TESgC)). An actual mean transmission efficiency was calculated for each F1 genotype (TEF1). The mean transmission efficiency for each of the parents (TESgF and TESgC) was calculated using only the data from the experiments a particular F1 was tested in and then the mean value for that F1 was corrected based on the parent values. If the mean transmission efficiency of the F1 genotype was lower than the mean for Sg-C, its transmission efficiency was 0%. Similarly, if the mean transmission efficiency of the F1 genotype was higher than the mean for Sg-F, its transmission efficiency was 100%. In most cases, the mean of the F1 genotype was between the mean values for the parents. The range of corrected transmission efficiencies of RPV and PAV for each F1 hybrid is shown in Figures 3 and 4.

**Figure 5.**
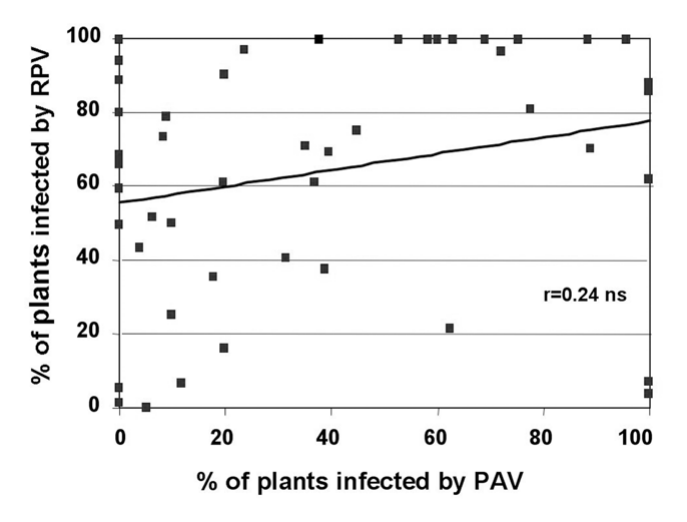
Correlation analysis of BYDV-PAV and CYDV-RPV transmission efficiency. Each data point represents a different clonal lineage and the line corresponds to the rcm of Via (1991).

F1 genotypes were genetically variable for transmission of PAV (F 58,86 = 5.08, P<0.001) and RPV (F 55,89 = 3.96, P<0.000), indicating that vector competence segregated in the F1 generation. This suggests that at least one of the parents, for at least one of the loci involved, was not fixed as the F1 generation would otherwise be homogeneous. This is not unusual when parental genotypes used for the crosses are drawn from a natural population. The broad-sense heritability (H2) was equal to 0.60 and 0.42 for PAV and RPV transmission, respectively, indicating that a significant proportion (60% and 42%) of the variation in the ability of F1 genotypes to transmit PAV and RPV was due to genetic differences between F1 genotypes. There was directional dominance found for transmission of both viruses, as F1 genotypes displayed an ability to transmit PAV (F1,169 = 0.18, P = 0.67) and RPV (F1,174 = 0.11, P = 0.73) with similar efficiencies as Sg-F. The mean transmission efficiencies for the F1 population were significantly higher than Sg-C for both PAV (F1,172 = 27.56, P<0.001) and RPV (F1,176 = 69.51, P<0.001) (Figure 1). There was a weak correlation between the ability to transmit RPV and PAV in the F1 genotypes (rcm = 0.24, P = 0.051, Figure 5), indicating the ability to efficiently transmit PAV does not necessarily indicate an ability to efficiently transmit RPV, or vice versa. At least one of the loci involved in PAV transmission and in RPV transmission is different and segregating in the F1 progeny.

**Figure 6.**
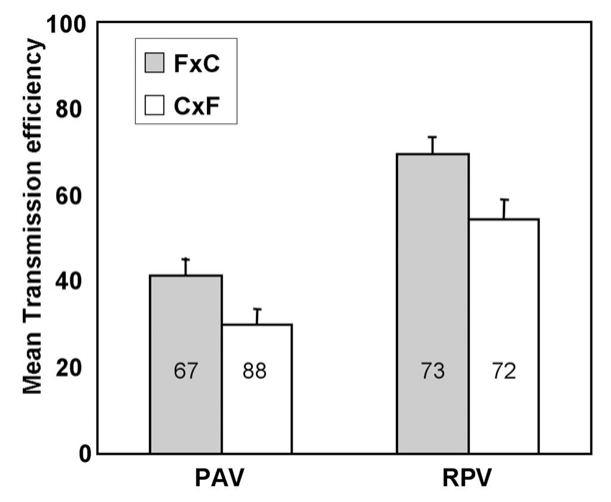
Mean transmission efficiency of BYDV-PAV and CYDV-RPV by the F1 genotypes from a reciprocal cross of Sg-C females and Sg-F males (C×F) or Sg-F females and Sg-C males (F×C). Transmisson efficency was calculated as the precentage of plants infested with viruliferous aphids that became infected following a 96 hr inoculation access period. The number of recipient plants used in each experiment was 12 to 16 and the number of experiments used to calculate the means and standard errors is shown in each bar.

Although the direction of the cross did not have a significant effect on the transmission efficiency of both PAV (F1,143 = 3.77; P = 0.054) or RPV (F 1,143 = 6.55, P = 0.12) (Figure 6), the F1 genotypes with Sg-F as the maternal parent were, on average, slightly more efficient vectors of PAV and RPV. This suggests that the maternally-inherited endosymbionts and/or cytoplasmic factors from the Sg-F genotype do not play a major role in the inheritance of virus transmission, but additional work is needed to definitively determine if they play any role.

## Discussion

The ability to generate sexuals and then maintain parthenogenetically reproducing hybrid aphid genotypes is a powerful tool for genetic analysis. We were able to maintain genetically stable genotypes and repeatedly assay each parent and hybrid genotype for multiple quantitative phenotypes. In purely sexual species, it is necessary to generate ”recombinant inbred lines” in order to obtain replication. However, inbreeding depression and residual genetic variation is common in recombinant inbred aphids (Via 1992), biasing genetic analyses. We acknowledge that clonal fidelity is not absolute and that genetic variation due to somatic and less often, germ line mutations does occur in clonal lineages of aphids (Loxdale and Lushai 2003; Shufran et al. 2003). Certainly, the clonal genotype is only as robust as the markers used to evaluate the stability of the clonal lineage. However, virus transmission phenotype has been a stable quantitative trait in aphid populations maintained in our laboratory for over 40 years (Power and Gray 1995). This level of genetic stability allows robust quantitative genetic studies on the mechanisms of virus transmission and a separation of the genetic and environmental factors that can influence the transmission process. Aphids offer unique opportunities for an in-depth genetic analysis of genetic traits such as vector competency, or more specifically, vector efficiency. Vector competency can be studied in other insects that reproduce sexually, however since isogenic populations are generally not available, transmission efficiency estimates can only be based on a genetically diverse population of insects. Therefore, it is not possible to separate genetic and environmental factors that influence transmission efficiency.

BYDV/CYDV is a major virus disease problem throughout the central United States where *S. graminum* is common. The role of *S. graminum* in the epidemiology of BYDV/CYDV in wheat is not well characterized in the central and western United States, although vector populations do exist in both regions (Halbert et al. 1992; Rayer et al. 2005). In the southeastern United States, *S. graminum* was found not to be a primary vector of BYDV/CYDV in winter wheat although it consistently colonizes wheat early in the growing season (Chapin et al. 2001; Gray et al. 1998). A nonvector population of *S. graminum* was characterized from wheat growing in South Carolina (Gray et al. 2002), whereas vector competent genotypes are common in this region colonizing wild grasses (Kelley 1994). New *S. graminum* biotypes are continually being recognized which cause injury to crops (Anstead et al. 2003). We speculated that the differential wheat cultiver data used to genotype new populations would predict vector competency. Although it appears that host preference is correlated to vector competence in *S. graminum* (Gray et al. 2002), virulence on differential wheat cultivars was not an accurate predictor of vector competence.

The influence of non-genetic factors are reflected in the variation in transmission efficiency of the parental genotypes over the 14-month period of the study. Heritability estimates attributed a significant amount of variation in transmission efficiency of F1 genotypes to genetic factors, but obviously other factors such as temperature, humidity, light, feeding duration, quality of virus source and recipient plants, and sites of aphid feeding have all been shown to influence transmission of BYDV/CYDV (Power and Gray 1995). These abiotic factors are undoubtedly contributing to the variation observed in this study. Although the acquisition and inoculation feeding periods were performed in temperature controlled growth rooms, the virus source plants and inoculated plants were maintained in greenhouses and subject to temperature fluctuations and occasional high temperatures. It is our experience that the transmission efficiency of BYDV/CYDV is generally lower at high temperatures (ie. the summer months or warm, sunny periods in spring and fell (Gray, unpublished data). This trend was evident in this study (Figure 2), and we attempted to control for environmental fluctuations by scaling the F1 transmission data using the parental phenotypes.

Repeated measurements of transmission efficiency in F1 genotypes allowed us to get a sense inheritance of the transmission efficiency phenotype. The parental aphids used in this study were not genetically fixed for transmission phenotype, as seen by segregation in the F1 generation. This is to be expected from any field-collected population, versus a population developed by inbreeding in the laboratory. We also noted that individual F1 genotypes transmitted BYDV or CYDV more efficiently than the vector parent. This indicates the parental vector genotype, Sg-F, was not homozygous for all genes facilitating virus transmission. Similar results were reported in previous studies using different aphid-virus systems (Burrows et al. 2006; Dedryver et al. 2005). The numbers of hybrids used in this study are too low to make any definitive conclusions regarding the genetics of virus transmission, but we can conclude from this and previous studies (Bjorling and Ossiannilsson 1958; Burrows et al. 2006; Dedryver et al. 2005; Papura et al. 2002) that the inheritance of virus transmission is a complex, genetically controlled phenotype involving multiple genes. Furthermore, the large number of F1 genotypes with measurable differences in the transmission efficiency of PAV and RPV indicate that not all the genes involved in transmission of PAV and RPV are shared. Similar results were found for the transmission of CYDV-RPV and BYDV-SGV by F1 and F2 hybrids derived from a different set of *S. graminum* parents (Burrows et al. 2006). Intuitively one might expect this to be the case due to the high degree of vector specificity previously reported for the viruses that cause barley yellow dwarf (Gildow 1993; Gildow and Gray 1993; Rochow 1969). Our data indicate that vector specificity is under genetic control.

Clearly, there are significant differences in the molecular recognition signals between the virus and the aphid that allow the virus to move across hindgut and accessory salivary gland tissues, and perhaps also for survival in the aphid hemolymph (see Gray and Gildow 2003 for review). Burrows et al. 2006 provided the definitive proof that hindgut and accessory salivary gland barriers to transmission of CYDV-RPV present in parental genotypes are genetically controlled by separate sets of genes that segregate in F2 progeny. Hemolymph-associated factors as barriers or facilitators of transmission of BYDV/CYDV in *S. graminum* remain uncertain. Symbionin, a protein produced in copious amounts by *Buchnera* sp. symbionts of aphids, including *S. graminum*, does bind to a domain of the minor structural protein of BYDV/CYDV *in vitro* and has been hypothesized to protect the virus from degradation in the aphid (van den Heuvel et al. 1999). However, symbionin does not influence the vector-specific transmission of BYDV/CYDV species (Van den Heuvel et al. 1997).

Furthermore, we have no data to indicate that symbionin plays a significant role in the lack of BYDV-PAV or CYDV-RPV transmission by Sg-SC. The direction of the cross was not a major factor in transmission efficiency (Figure 6), suggesting that endosymbionts inherited from the maternal parent are not playing a significant role in BYDV/CYDV transmission by *S. graminum*. Burrows et al. (2006) found that viral RNA was stable in the hemolymph of vector and nonvector *S. graminum* genotypes. Also, there were no conserved differences in symbionin sequence from vector and nonvector genotypes of *R. padi* and *S. graminum* (Gray, unpublished data). All of this leads us to believe that symbionin plays a minor, if any, role in genetic regulation of BYDV/CYDV transmission by *S. graminum*.

The ability to generate and maintain stable genotypes of vector and nonvector aphids within the same species will allow continued study on the mechanisms of circulative virus transmission. Burrows et al. 2006 have identified individual progeny of a cross between vector and nonvector genotypes of *S. graminum* that differ in where the barrier to transmission (hindgut or accessory salivary gland) is located. These aphid genotypes will be invaluable in studies aimed at identifying genes and gene products that are involved in regulating the movement of virus across specific tissues in the aphid. Coupled with other technologies to study the genomics and proteomics of virus transmission, and information being developed on virus binding proteins in aphids (Li et al. 2001; Seddas et al. 2004), genetic systems such as the one described in this paper will facilitate the identification of insect genes regulating virus transmission. This knowledge will aid in the development of novel control strategies for all type of insect transmitted pathogens.

## Abbreviations

BYDV-PAV: Barley yellow dwarf virus-PAV
CYDV-RPV: Cereal yellow dwarf virus-RPV
Sg-C: nonvector biotype of Schizaphis graminum
Sg-Fvector: biotype of Schizaphis graminum
